# Imputation and Reanalysis of ExomeChip Data Identifies Novel, Conditional and Joint Genetic Effects on Parkinson’s Disease Risk

**DOI:** 10.3390/genes12050689

**Published:** 2021-05-04

**Authors:** Linduni M. Rodrigo, Dale R. Nyholt

**Affiliations:** School of Biomedical Sciences, Faculty of Health, Centre for Genomics and Personalised Health, Queensland University of Technology, Brisbane, QLD 4000, Australia; linduni06@gmail.com

**Keywords:** Parkinson’s disease, genotype imputation, GWAS, SNP–SNP interactions, machine learning

## Abstract

Given that improved imputation software and high-coverage whole genome sequence (WGS)-based haplotype reference panels now enable inexpensive approximation of WGS genotype data, we hypothesised that WGS-based imputation and analysis of existing ExomeChip-based genome-wide association (GWA) data will identify novel intronic and intergenic single nucleotide polymorphism (SNP) effects associated with complex disease risk. In this study, we reanalysed a Parkinson’s disease (PD) dataset comprising 5540 cases and 5862 controls genotyped using the ExomeChip-based NeuroX array. After genotype imputation and extensive quality control, GWA analysis was performed using PLINK and a recently developed machine learning approach (GenEpi), to identify novel, conditional and joint genetic effects associated with PD. In addition to improved validation of previously reported loci, we identified five novel genome-wide significant loci associated with PD: three (rs137887044, rs78837976 and rs117672332) with 0.01 < MAF < 0.05, and two (rs187989831 and rs12100172) with MAF < 0.01. Conditional analysis within genome-wide significant loci revealed four loci (*p* < 1 × 10^−5^) with multiple independent risk variants, while GenEpi analysis identified SNP–SNP interactions in seven genes. In addition to identifying novel risk loci for PD, these results demonstrate that WGS-based imputation and analysis of existing exome genotype data can identify novel intronic and intergenic SNP effects associated with complex disease risk.

## 1. Introduction

Over the past decade, genome-wide association studies (GWAS) have successfully identified many individual common genetic variants (i.e., single nucleotide polymorphisms (SNPs)) associated with the risk of a wide range of complex diseases. However, due to insufficient statistical power, the genetic effects identified by typical GWAS studies tend to explain only a small fraction of the overall genetic variation underlying complex diseases [1]. In order to identify this missing heritability of complex diseases, it is important to explore the role of low-frequency SNPs: SNPs with minor allele frequency (MAF) less than 0.05 at novel or established risk loci and the potential interaction between SNPs that might have a strong contribution towards disease risk compared to their main effects. However, because most GWAS studies focus on generating genetic data in new samples and use standard statistical tools to detect common SNPs with marginal effects, they do not identify heterogeneous effects or epistasis interaction effects of multiple SNPs.

Next-generation sequencing (NGS) technology allowed the development and use of cost-effective genotyping arrays to efficiently genotype and assess common genome-wide genetic variation in large samples, leading to the discovery of thousands of risk SNPs for many complex diseases. The genetic resolution of those large genotyped datasets can be increased via imputation of unobserved common and rare variants with advanced genotype imputation software which use new dense whole genome sequence (WGS)-based haplotype reference panels [2]. In addition, new methodologies have been recently developed using machine learning approaches to efficiently discover joint genetic effects of variants contributing towards complex disease risk by tackling the challenges of traditional GWAS, i.e., low statistical power to detect conditional and interaction effects due to the high dimensionality and multiple-test burden [3].

Although the vast majority of GWAS utilise common SNP arrays, some GWAS utilised exome-based arrays, such as the Ilumina HumanExome BeadChip (ExomeChip), to test ~240,000 mostly nonsynonymous coding variants across the human genome. Given that improved imputation software and WGS-based haplotype reference panels now enable inexpensive approximation of WGS genotype data, we hypothesised that WGS-based imputation and analysis of existing ExomeChip-based GWAS data will identify novel intronic and intergenic SNP effects associated with complex disease risk. We also hypothesised that analysis of imputed data using machine learning will identify novel, conditional and joint effects of both common and/or rare SNPs related to complex disease risk. To test our hypothesis, this study focuses on Parkinson’s disease (PD), a common neurodegenerative disorder with a complex genetic component mainly affecting adults aged over 60 years [4]. PD exerts a substantial burden to the global health and economy, and is expected to affect more than 12 million people by 2040 worldwide. During the past two decades, numerous GWAS have been conducted to understand the pathophysiology of PD and they have identified over 90 independent risk variants, of which most are common, explaining a heritability of 16–36% [5]; however, the majority of the genetic heritability of PD remains to be uncovered. Furthermore, the role of rare variants and the interaction effects of both common and rare variants are yet to be explored.

We accessed a large powerful PD NeuroX dataset available in dbGaP (dbGaP Study Accession: phs000918.v1.p1) that comprises 5540 cases and 5862 controls of European ancestry and first applied careful quality control procedures. Then, the dense WGS-based Haplotype Reference Consortium (HRC) reference panel was used to perform genotype imputation followed by standard GWAS association analysis, conditional analysis and interaction analysis using PLINK and R. Lastly, the recently published python package—GenEpi: a machine learning approach for gene-based epistasis discovery [3]—was utilised to identify joint genetic effects associated with PD.

## 2. Materials and Methods

### 2.1. Data

This study utilised the individual-level PD case-control dataset available in dbGaP (https://www.ncbi.nlm.nih.gov/projects/gap/cgi-bin/study.cgi?study_id=phs000918.v1.p1, accessed on 15 August 2019) which was first used to replicate the findings of a PD meta-analysis [6]. The PD dataset comprises 5540 cases and 5862 controls (6842 males and 4560 females) of European ancestry genotyped using the Illumina NeuroX genotyping array. This is a combination of the Illumina HumanExome BeadChip (ExomeChip) array, v1.1 (242,901 variants) and custom content (24,706 variants) focused on neurodegenerative diseases. The exome array primarily (90%) contains nonsynonymous with a high proportion of rare variants (82% with MAF < 0.01) and the majority (60%) of variants within the custom content are common (MAF > 0.05). The custom array also contains rare sequence-based variants from familial studies that are not available in the 1000 Genomes and NHLBI’s Exome Sequencing projects and rare sequence-based variants (frequency less than 0.05 in the population used) from cohort studies focused on neurodegenerative disorders including PD [7].

### 2.2. Quality Control (QC)

Quality control for this study was performed using PLINK v1.9 [8] and recommended protocols [9,10,11], with a few modifications to account for the customized content of the NeuroX array. First, individuals with call rates below 95%, as well as individuals with gender discrepancy (individuals with genetically predicted and reported sex difference) and individuals showing excess heterozygosity (deviate ±3 SD from sample heterozygosity rate) were excluded. After this initial individual filtering, SNPs with per SNP missingness greater than 5% as well as SNPs with MAC below 3 and SNPs that are not in Hardy–Weinberg equilibrium (HWE *p*-value ˂ 1 × 10^−6^) were removed. Then, the pairwise identity by descent (IBD) was calculated using PLINK v1.9 after pruning for linkage disequilibrium (LD), where SNPs with *r*^2^ > 0.02 within 50-SNP sliding window were pruned out, and used to remove cryptically related individuals (individuals with lowest call rate from the pairs of individuals with ‘pi_hat’ > 0.2). Finally, principal component analysis (PCA) was used to identify and exclude individuals with genetic ancestry inconsistent with European descent compared to the 1000 G reference panel.

### 2.3. Imputation and Post Imputation QC

Genotype imputation using the HRC reference panel (version r1.1, which consists of 64,940 haplotypes of predominantly European ancestry) was performed with minimac4 using next-generation genotype imputation service and methods [12] available in Michigan Imputation Server (https://imputationserver.sph.umich.edu/index.html accessed on 18 January 2020). Input data preparation for imputation according the data preparation guidelines provided by Michigan Imputation Server and post-imputation QC was done using PLINK v2.0 and vcftools [13]. Quality control for HRC reference as a pre-preparation step was carried out using the toolbox provided by Will Rayner (http://www.well.ox.ac.uk/~wrayner/tools/ accessed on 21 January 2020). Genotype imputation for each chromosome was performed after several QC and phasing steps by the server and imputed data which includes dose.vcf and info file for each chromosome were download directly from the server. The imputation quality of the imputed SNPs was evaluated using minimac4 info score provided in .info file. Post-imputation QC was done by extracting SNPs with MAF ≥ 0.001, info score ≥ 0.5, and HWE *p*-value ≥ 1 × 10^−7^ in controls, to identify quality SNPs for further analysis. Using PLINK, the imputed genotype posterior probabilities in the VCF files were converted to Oxford-format (.gen) best-guess genotypes for the GenEpi interaction analyses.

### 2.4. Association Analysis and Conditional Analysis

Association analysis of imputed genotype dosage data was done using PLINK v2.0. First, PCA was carried out with LD pruned SNPs (following the same criteria described in QC section) to generate eigenvectors. Then, logistic regression was performed, adjusting for the first two principal components (PCs), age, and sex to examine the additive effect for each SNP on PD risk.

Conditional analyses were next applied to identify secondary association signals. For each genome-wide significant locus identified in the association analysis, we performed region-wise conditional analysis using PLINK and tested all the SNPs in the region while adjusting for the most significant (“index”) SNP in that region, as well as for all the covariates analogous to the association analysis.

### 2.5. Interaction Analysis

To identify joint effects of SNPs on PD risk, we used a recently developed computational package called GenEpi [3], which applies a gene-based machine learning approach to discover pair-wise epistasis associated with a phenotype. In GenEpi, the first step is to group genetic variants by a set of loci (i.e., genes) in the genome using gene information available in the UCSC human genome annotation database [14] followed by dimensionality reduction of genetic features in each locus using LD which involves grouping of features into LD blocks using a given *r^2^* and *D*’ threshold and selection of the features with the largest MAF to represent each block. The selected genotype features of each single gene will then be independently modelled by L1-regularised regression. In the next stage, to identify cross-gene epistasis features, both the individual SNPs and the previously selected within-gene epistasis features are pooled together and used in L1-regularised regression to select the final genotype feature set. In addition, users have the option to include environmental factors to build a final model. Evaluation of the final model is available in the design by 2-fold cross validation (CV). Given this study’s focus is to identify SNP-SNP interactions associated with PD risk (not prediction), SNP–SNP interactions were further analysed by generating counts and frequencies of each two-locus genotype using PLINK v1.9 to understand the manner of each interaction.

For the current study, GenEpi was applied to best-guess genotypes on the set of SNPs with nominal statistically significant association results (*p*-value < 0.05) using the thresholds of *D*’  >  0.8 and *r*^2^  >  0.8 to generate LD blocks of features, and the first two ancestry PCs, age and sex, as environmental factors.

## 3. Results

### 3.1. Genome-Wide Association Analysis

In the current study, the initial PD case-control GWAS individual-level dataset downloaded from dbGaP was quality controlled to exclude low-quality variants and samples (see Supplementary Table S1 for composition of the sample) using customised quality control procedures (Methods and Supplementary Table S2) to include low-frequency variants for further analysis. After quality control steps, a total of 10,533 individuals (5167 cases, 5366 controls) and 110,504 SNPs remained for genotype imputation.

After filtering out low-quality individuals and SNPs, the remaining dataset was processed following the data preparation guidelines provided by the Michigan Imputation Server. Briefly, for each chromosome, VCF files created using VCFCooker were sorted by genome position and uploaded as input files to the server. The server’s imputation process, including pre-phrasing and imputation to the HRC reference panel using minimac4, took about 15 h after successful input validation and quality control. The total imputed dataset downloaded from the server contained approximately 40 million SNPs. Post-imputation quality screening using minimac4 info score, MAF and HWE *p*-value as parameters resulted in a substantially increased dataset. Compared to the original NeuroX genotyped dataset, the final imputed dataset contained 1,465,938 SNPs with good imputation quality (minimac4 info score ≥ 0.5, MAF ≥ 0.001, and HWE *p*-value ≥ 1 × 10^−7^ in controls), representing an increase of 1200%. Among the imputed SNPs, 733,576 were common (MAF ≥ 0.05) and 732,362 were low frequency (MAF ˂ 0.05). Moreover, despite the vast majority (73%) of SNPs in the initial NeuroX dataset being exonic, many intronic and intergenic SNPs were imputed; indeed, the imputed dataset comprised 53% intronic, 40% intergenic and 7% exonic SNPs (Table 1 and Supplementary Table S5).

**Figure 1:**
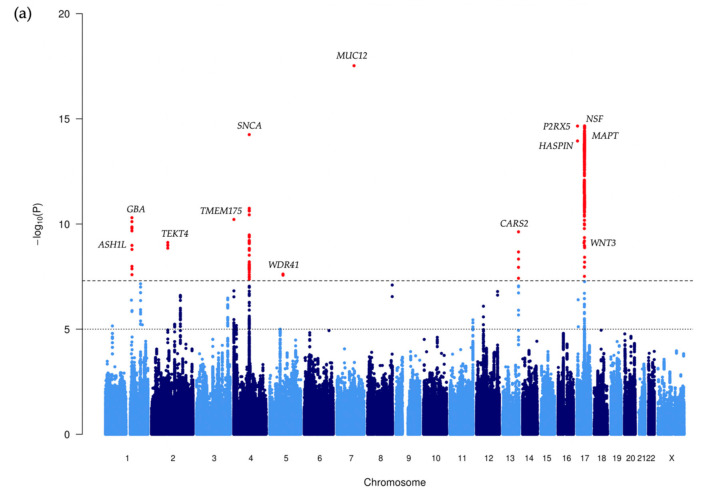

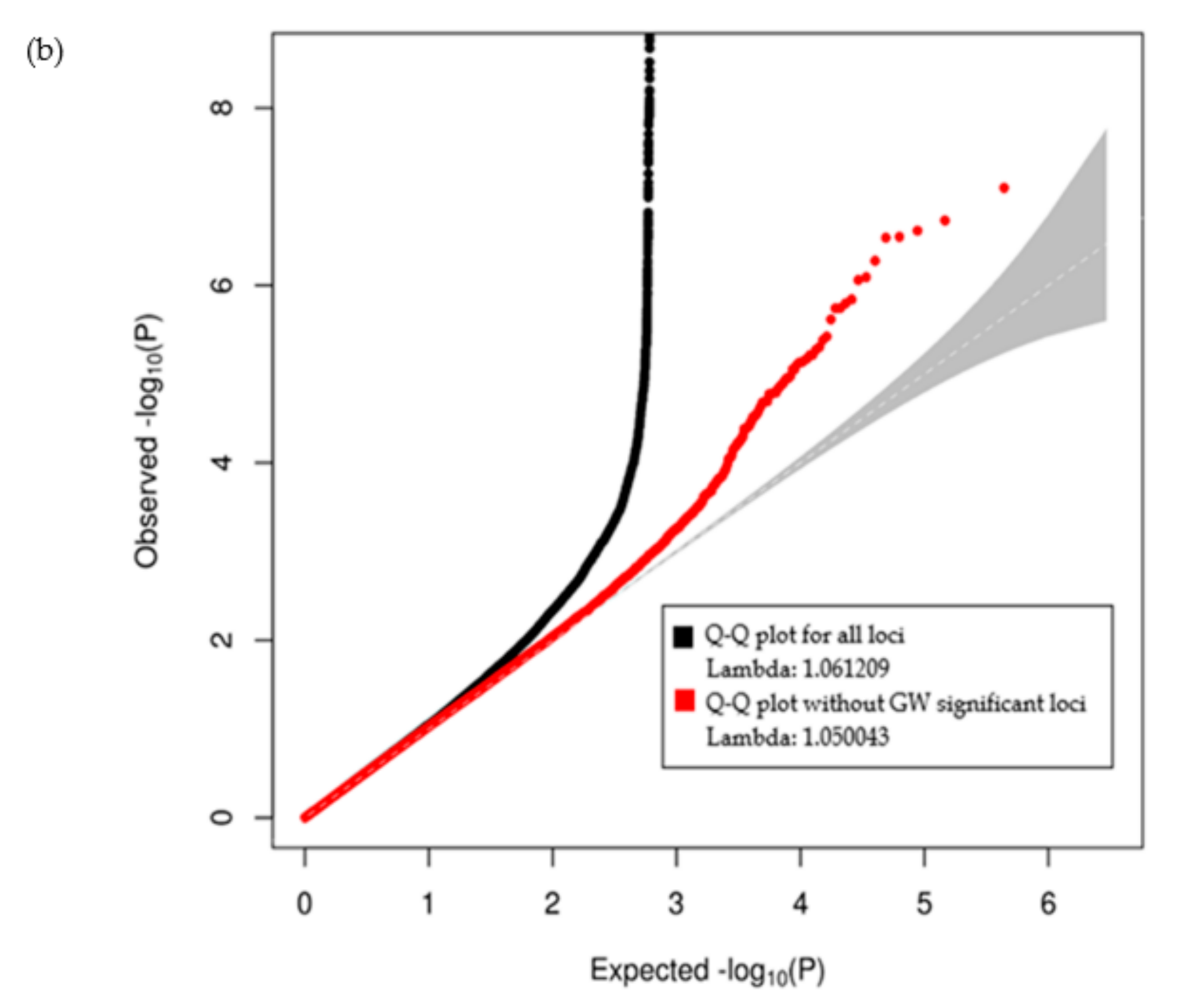
(**a**) **Manhattan and** (**b**) **Q–Q plot of the genome-wide association analysis.** The Manhattan plot, representing the −log_10_ *p*-values against the chromosome position. All genome-wide (GW) significant SNPs are depicted in red and the nearest gene of the most significant variant in each locus is labelled. The Q–Q plot shows the expected −log_10_ *p*-values under the null hypothesis on the x axis, while observed −log10 *p*-values are represented on the y axis. The λ is a measure of the genomic inflation (observed median χ^2^ test statistic/median expected χ^2^ test statistic under the null hypothesis).

**Figure 2:**
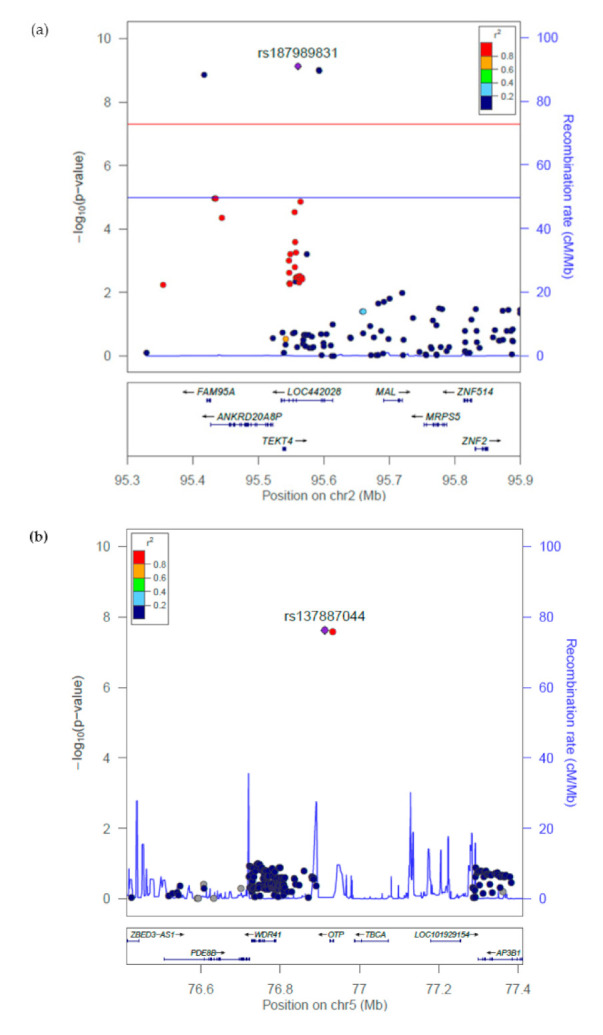

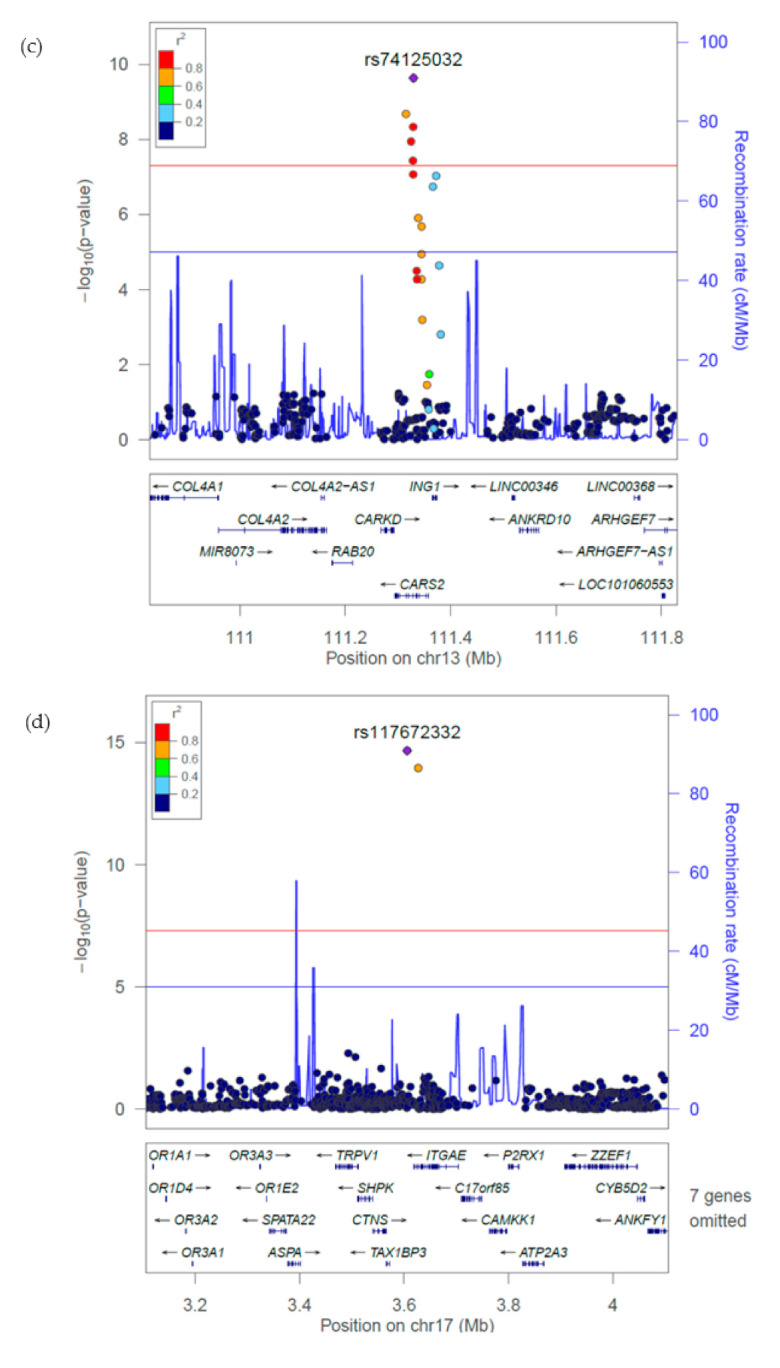

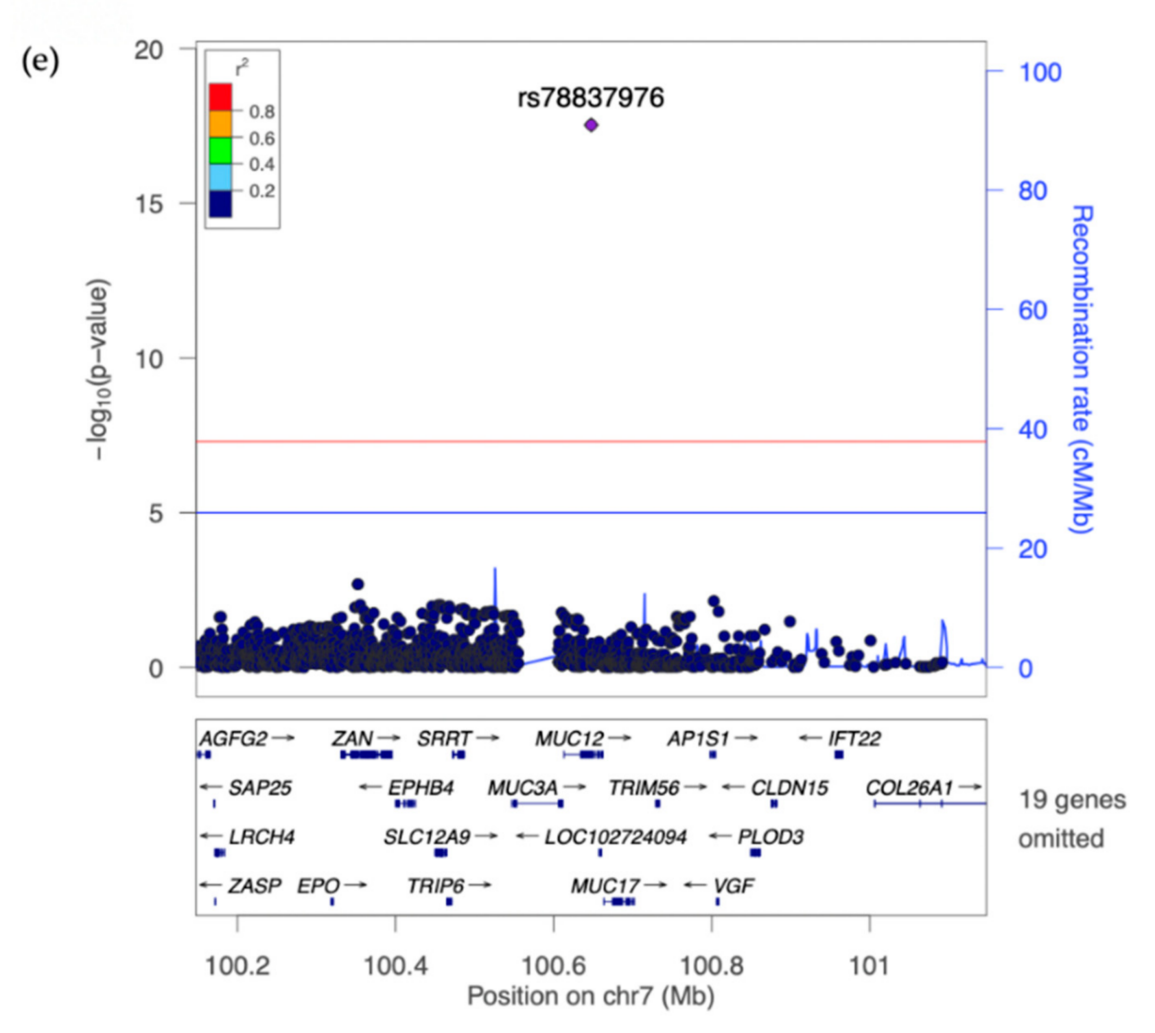
**LocusZoom plots of novel genome-wide significant PD loci.** (**a**) rs187989831 near *TEKT4* on 2q11.1, (**b**) rs137887044 near *WDR41* on 5q14.1, (**c**) rs74125032 in *CARS2* on 13q34, (**d**) rs117672332 in *ITGAE/HASPIN* on 17p13.2, and (**e**) rs78837976 in *MUC12* on 7q22.1. Association significance with PD is shown as −log_10_ *p*-values on the left *y*-axis. The most significant SNP represented by purple colour diamond. All other SNPs are shown as circles and are colour coded according to the strength of LD with the most significant SNP (LD measured using the European 1000 Genomes data).

After testing each SNP for association with PD risk using logistic regression including age, sex and the first two ancestry PCs as covariates (Methods), association results were obtained for 1,465,918 SNPs, with 20 very rare variants producing NA values where the logistic regression failed to converge (see Supplementary Data S1 ‘NeuroX_Reanalysis_Summary_Statistics.txt’ and Supplementary Note S1 ‘Description_NeuroX_Reanalysis_Summary_Statistics.txt’), A total of 11 independent association signals for PD were identified, reaching a genome-wide significant *p*-value (*p* ≤ 5 × 10^−8^), including 5 newly identified signals that are more than 1 MB from the previously reported PD risk loci (Table 2, Figure 1, Supplementary Table S3, Supplementary Figure S2). Of these novel loci, three are driven by low-frequency (0.01 < MAF < 0.05) variants (rs137887044 in WDR41 on chromosome 5q14.1, rs78837976 in MUC12 on 7q22.1, and rs117672332 in ITGAE/HASPIN on 17p13.2), and two by rare (MAF < 0.01) variants (rs187989831 near TEKT4 on chromosome 2q11.1 and rs12100172 in CARS2 on 13q34). LocusZoom plots of the identified novel loci are shown in Figure 2.

Overall, with this individual SNP analysis of the imputed data, we were able to identify seven PD risk loci that were not reported in the original Nalls et al. (2014) study, comprising five novel loci and two other loci: rs983361 in SNCA at 4q22.1, which has been reported to be associated with PD age at onset [15] and rs7221167 in MAPT at 17q21.31, which has been reported but failed final filtering and QC in Nalls et al. 2019 PD GWAS [5] (Table 2).

Conditional analysis revealed nine loci with more than one independent risk signal, including two loci (within *SNCA* and *HASPIN*) reaching genome-wide significance (*p <* 5 × 10^−8^). Of those, one secondary association signal is in the newly identified gene *HASPIN* (rs11653889 and rs117672332 at 17p13.2) and loci within *GBA*, *TMEM175*, *SNCA*, and *GAK/DGKQ* had been previously identified as multi-signal loci by PD GWAS. In addition, of those secondary association signals identified in conditional analysis, four (rs113319394, rs3806789, rs74125084 and rs11653889) have high LD (*r*^2^ > 0.1) with the index SNP and five (rs112344141, rs181580861, rs72765119, rs28645997, rs3851784) have very low LD (*r*^2^ ≤ 0.01) with the index SNP. The locus with a secondary association signal at 4q22.1 (rs3806789 in *SNCA*) showed the largest decrease in *p*-value (from 2.10 × 10^−2^ to 9.13 × 10^−10^) producing a conditional odds ratio of 1.2 when conditioned on rs356182, indicating significant allelic heterogeneity at this locus. Detailed summary statistics on all nine secondary loci can be found in the Table 3 (LocusZoom plots of these loci are available in Supplementary Figure S1).

### 3.2. Comparison of Association Results with Nalls et al. (2014) Findings

The NeuroX dataset has been previously used by Nalls et al. (2014) to replicate 26 SNP loci found to be associated with PD disease risk (*p* < 5 × 10^−8^) from a meta-analysis of genome-wide association data (Discovery Phase). Of these 26 SNPs, eight were not available in the NeuroX dataset for analysis due to failed assay design or quality control, so for the replication study, a suitable proxy SNP was selected. Of the 26 PD risk loci examined in the original NeuroX study by Nalls et al. (2014), 18 were replicated (*p* < 0.05) using the same SNP and an additional four loci were replicated using proxy SNPs.

In the current study, of the eight SNPs missing in the NeuroX dataset, apart from one SNP (rs8118008)—due to its absence in the HRC reference panel—seven were successfully imputed. Analysis of the imputed genotype data successfully replicated 21 of the 22 PD risk loci that were originally replicated in Nalls et al. (2014), including the rs8118008 locus that although not imputed itself, was replicated using a stronger proxy SNP rs8125675 (*r^2^* = 1) compared to the proxy SNP (rs55785911, *r^2^* = 0.85) used in Nalls et al. (2014). Notably, our analysis was able to impute and replicate (*p* = 0.031) an additional PD risk locus (rs62120679) that was not replicated using a moderate (*r^2^* = 0.49) proxy SNP (rs10402629) in Nalls et al. (2014) (Table 4). In contrast, one original SNP (rs11158026) replicated by Nalls et al. (2014) with *p* = 0.039 was not replicated in our imputed dataset (*p* = 0.186). Indeed, prior to imputation, analysis of our QC’d NeuroX genotypes produced a *p*-value of 0.119 for rs11158026, indicating the difference in replication was due to the additional samples used in our analyses (i.e., 5353 cases and 5551 controls in Nalls et al. (2014) compared to 5540 cases and 5862 controls in the current study) (Table 4 and Supplementary Table S4).

### 3.3. Novel Low-Frequency Variants Associated with PD

Along with those known genetic loci associated with PD, we also identified five novel loci, of which three were driven by low-frequency variants with effect sizes (OR > 1.85; Table 2, Figure 1). One of these loci, driven by a low-frequency intron variant in WDR41 gene at chromosome 5q14.1 (rs137887044, OR = 1.850 [1.489–2.295], *p* = 2.41 × 10^−8^), has previously been implicated in multiple neurological disorders. The LocusZoom plot for the 1 Mb region of this novel SNP (Figure 2b), showed another genome-wide significant SNP (rs148662448 near WDR41) having strong LD (*r^2^* = 0.899) with the novel SNP rs137887044. As expected, when conditioned on rs137887044, rs148662448 no longer showed evidence for association (*p* = 0.411), indicating a single genetic risk factor exists at this location.

Our analysis highlighted two novel rare variants. One at chromosome 2q11.1(rs187989831, *p* = 7.56 × 10^−10^) near TEKT4. As shown in the LocusZoom plot (Figure 2a), there are two other genome-wide significant SNPs (rs1281734107 and rs78890475) in low LD (*r^2^* ≤ 0.2) close to the novel index SNP. However, conditional analyses conditioning on rs187989831 found only weak evidence for residual association (0.005 < *p* < 0.03) of these two SNPs at this locus. The second rare novel variant (rs74125032, *p* = 2.15 × 10^−10^) lies within an intron of the CARS2 gene on chromosome 13q34. However, these two variants have extremely small OR in this study and the reason could be that these variants are extremely rare and have very low genotype frequency within the NeuroX dataset.

### 3.4. Joint Genetic Effects on PD Risk

**Figure 3:**
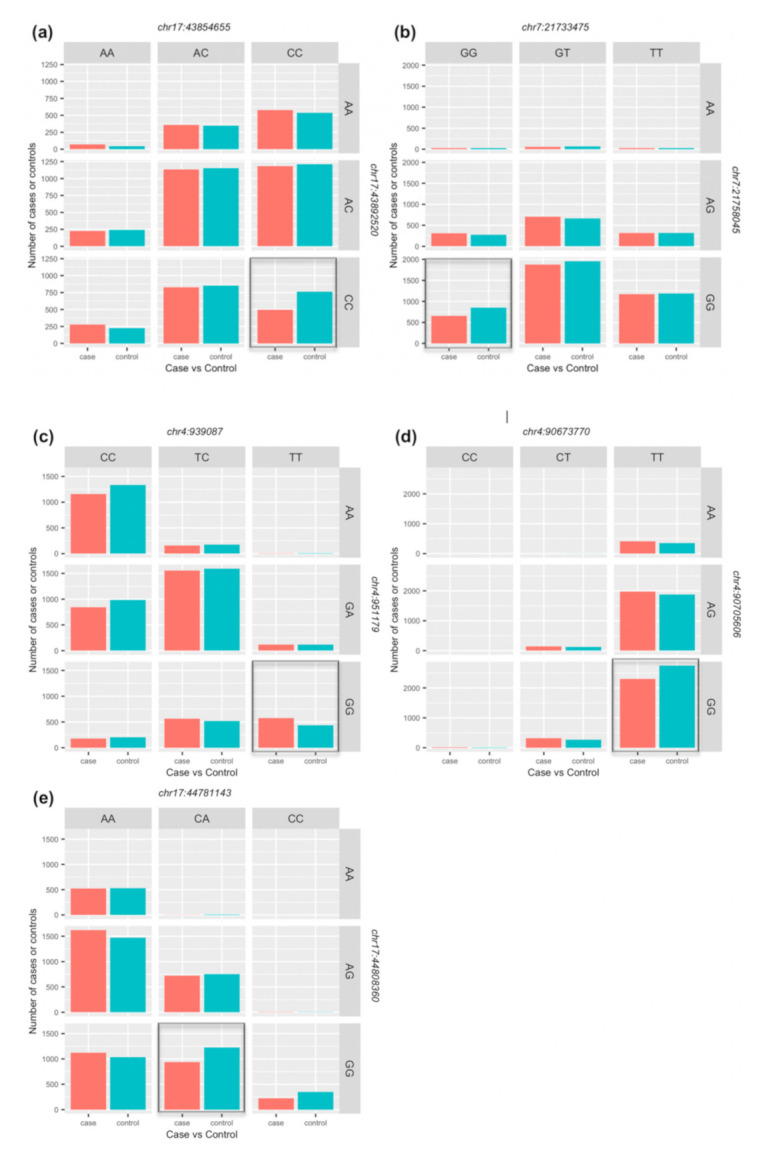
**Genotype frequency for the combination of SNPs identified by GenEpi.** (**a**) Frequency in cases and controls of each genotype combination of the most significant SNP–SNP interaction effect on PD; (**b**) Frequency in cases and controls of each genotype combination of the SNP–SNP interaction effect identified in novel PD risk loci. (**c**–**e**) show frequency differences in cases and controls for each genotype combination of the most significant SNP–SNP interactions in other three independent loci. The dark-shaded cell of each figure represents the combination that has the strongest effect.

Machine learning (GenEpi) association analyses identified significant (*p* < 3.77 × 10^−6^) SNP–SNP interactions at five independent genomic loci harbouring eight different genes (Table 5). Seven of the eight genes (GAK, TMEM175, SNCA, PLEKHM1, CRHR1, MAPT and NSF) have been implicated via GWAS by others as having individual SNPs associated with PD risk, whereas a joint effect of two SNPs at chromosome 7p15.3 (rs2965400 and rs6461595, *p* = 3.77 × 10^−6^) within an intron of the DNAH11 gene has not previously been implicated in PD, although it has been reported to be associated with cholesterol level and (age-related) cognitive decline. Furthermore, the most significant interaction effect on PD was found between two SNPs rs34186148 and rs242941 (*p* = 4.78 × 10^−10^) at chromosome 17q21.31 in the CRHR1 gene with the homozygous CC genotype being protective for PD at both SNPs. The protein coding CRHR1 gene is reported to be associated with anxiety and depression which are common in PD. Figure 3 shows the genotype combination of SNPs in CRHR1 (Figure 3a), DNAH11 (Figure 3b) and the most significant interaction in other three independent loci (TMEM175 at 4p16.3, SNCA at 4q22.1 and NSF at 17q21.31), highlighting frequency differences in cases and controls for different genotype combinations underlying the significant association with PD.

## 4. Discussion

In this study, we reanalysed an ExomeChip-based NeuroX dataset—previously used for the replication of GWA meta-analysis results [6,16,17]—to identify novel common and rare SNPs and their interactions associated with PD risk. Starting with only 110,504 NeuroX SNPs passing QC, comprising predominantly (73%) exonic and less common variants, we accurately imputed 1,465,938 SNPs using the HRC reference panel. The imputed dataset comprised 53% intronic, 40% intergenic and 7% exonic SNPs and spanned a wide frequency range including rarer as well as more common SNPs across chromosomes 1–22 and X. A review of the literature only found examples focussing on genome-wide imputation of exonic variants. For example, Auer et al. (2012) performed genotype imputation of exome sequence variants in a sample of more than 13,000 African Americans with Affymetrix GWA genotyping array (Affy6.0) data, using a reference comprising 761 African Americans with both Affy6.0 genotype data (838,337 SNPs with MAF > 0.01 spread across the genome) and exome sequence data to identify exonic variants associated with blood cell counts [18]. Similar studies in the same cohort were performed by Johnsen et al. (2013) and Du et al. (2014) to identify novel low-frequency variants that contribute to von Willebrand factor [19] and adult body height [20]. In contrast, we imputed common and rare variants across the genome using a WGS-based HRC reference panel, starting with predominantly rare exonic variants. In the current study, compared to the association results from the original NeuroX dataset, the results after imputation produced (i) more robust evidence for replication with smaller *p*-values for most of the original significant SNPs, and (ii) a larger number of genome-wide significant loci associated with PD.

Association analysis of imputed genetic data confirmed several already-known PD risk loci and also allowed us to identify five novel association signals driven by low-frequency variants in or near *TEKT4, WDR41, MUC12, CARS2,* and *ITGAE*/*HASPIN*. Of those, first, the low-frequency variant identified in *WDR41* at chromosome 5q14.1 showed a near two-fold increased risk for PD and *WDR41* which is associated with several neurogenerative disorders and could be a potential candidate gene to identify PD risk. *WDR41* is a protein-coding gene and diseases associated with this gene include striatal degeneration, autosomal dominant 1, a rare autosomal-dominant movement disorder with some motor symptoms similar to PD, and frontotemporal dementia and/or amyotrophic lateral sclerosis 1, an autosomal dominant neurodegenerative disorder [21]. Importantly, several variants in *WDR41* have been identified in previous GWAS having near genome-wide significant association signals for: Alzheimer’s disease (AD) (*p* = 7 × 10^−7^) [22]; caudate nucleus volume (*p* = 2 × 10^−7^), where caudate is a subcortical brain structure implicated in many common neurological and psychiatric disorders [23]; and epileptogenesis (*p* = 5 × 10^−6^) [24] in European populations. AD is also an age-related neurodegenerative condition caused by damaged brain cells and both PD and AD can involve common symptoms such as anxiety, depression, and sleep disturbances; some studies have noted shared risk variants across AD and PD [25]. However, none of these studies were able to identify the same rare cording variants for both diseases, perhaps due to limited sample sizes and different data processing methods. These previous findings and results of the current study suggest that *WDR41* is a strong candidate gene involved in PD risk.

Second, the variant near *TEKT4* is a very rare variant and thus showed an extreme odds ratio in the NeuroX sample. Such an extreme effect estimates that less common or rare genetic variants have large standard errors and result from the small number of alleles observed in the analysed case and control samples. Therefore, analyses in larger samples are required to produce more accurate effect estimates. That said, the near-QC threshold minimac info scores for the variants producing extreme OR values in this study (*r^2^* = 0.5334 for rs187989831 and *r^2^* = 0.50635 for rs74125032) could indicate that lower imputation quality may negatively influence the association test and effect estimation. Indeed, there are several challenges associated with both imputing and analysing rare genetic variants due to the low frequency of those variants in the study sample due to their low correlation with surrounding variants, especially compared to and with common genetic variants. Hence, replication via direct genotyping ideally in larger sample sizes is required to ultimately validate such findings.

Although the *TEKT4*-associated variant is rare and requires validation, it may be an important finding due to its potential involvement in sudden unexplained death in PD or seizure. Diseases potentially related to *TEKT4* include myoclonic juvenile epilepsy [26], a condition characterised by recurrent seizures which cause rapid, uncontrolled muscle jerks, muscle rigidity, convulsions, and loss of consciousness.

Of the genes implicated by the other novel SNP loci, *CARS2* is associated with combined oxidative phosphorylation deficiency and ovarian cancer. It was found that mutations in *CARS2* are associated with progressive myoclonus epilepsy [27] and could lead to a severe epileptic encephalopathy and complex movement disorder [28]. Epilepsy is an uncommon comorbidity of PD. Although rare, the coexistence of epilepsy and PD may influence PD progression [29]. Gruntz et al. (2018) clearly suggest that incident PD is associated with an increased incident epileptic seizures risk [30]. This suggests that these two rare variants could be possible candidate genes for PD risk and since epilepsy is associated with increased risk of sudden unexplained death in epilepsy [31], having variants at these loci, patients with increased risk of PD may experience sudden unexpected death. However, these two variants have extremely large odds ratios in this study, perhaps due to their very low genotype frequency within the analysed dataset. *MUC12* is associated with Tn polyagglutination syndrome and colorectal cancer and previous GWAS have pointed out the effect of the genetic variants in *MUC12* on hemoglobin levels and *CARS2* on diastolic blood pressure. However, there is no disease reported to be associated with the *HASPIN* gene, making it an important gene for further analysis.

In addition, our results show strong evidence for multiple association signals: one at chromosome 17p13.2 in *HASPIN* substantiating the importance of this gene in PD risk, and one at chromosome 4q22.1 in *SNCA.* SNPs at chromosome 4p22.1 are well known for their association with PD [15,32,33,34] and several other diseases including dementia with Lewy bodies [35].

Notably, this analysis using genotype imputation identified eight PD risk loci, including the five novel genetic loci mentioned above and two other loci in *SNCA* and *MAPT* that have been previously reported in other PD GWAS, that were not reported in the original Nalls et al. (2014) study which used the same NeuroX dataset to replicate their discovery phase findings without genotype imputation. Of these risk loci, all genetic variants in the five novel loci are low-frequency or rare variants, while variants in the two other previously reported loci are common genetic variants. In addition to the replication of PD risk loci identified and replicated in Nalls et al. (2014), our analysis was able to impute and replicate (*p* = 0.031) an additional PD risk locus (rs62120679 in *SPPL2B*) that was not replicated using a proxy SNP (rs10402629) in Nalls et al. (2014). These results support the utility of genotype imputation using dense reference panel such as HRC to assess genetic variants with wide frequency range.

Interestingly, our results provide support for the findings of a recent meta-analysis of whole-exome sequencing data by Gaare et al. (2020) [16] that was replicated using a cohort genotyped using the NeuroX array. This 2020 study found no evidence of rare mutation enrichment in genes within PD-associated loci. Similarly, our study found genome-wide significant associations of rare SNPs only within novel PD risk loci and not within known PD-associated loci.

The interaction analysis using the GenEpi machine learning approach identified eight SNP pairs having joint genetic effects associated with PD, including a strong genome-wide significant interaction association signal at chromosome 17q21.31 in *CRHR1*, although producing no significant association signals of those two SNPs individually for PD risk. Given that SNPs in *CRHR1* have been previously reported to be associated with PD [5,32,36,37] and Alzheimer’s disease [38] and SNP–SNP interactions are identified in *SNCA*, *GAK* and *MAPT*, a well-known risk gene for PD, this suggests that these joint effects are true findings and nicely demonstrate the utility of our approach to identify joint genetic effects associated with complex diseases like PD. However, in GenEpi two criteria were adopted before modelling the genotype features: first, exclude features with genotype frequency (proportion of a genotype among the total samples in the dataset) ≤ 5%; and second, exclude features with weak association (χ^2^ test *p* ≥ 0.01) with the disease. This limits the discovery of joint genetic effects of SNPs having relatively small main effects and the interactions of non-common SNPs.

Overall, the novel individual association signals in *TEKT4* and *WDR41* and the SNP–SNP interaction effect in *CRHR1* identified in this study are important because although *TEKT4* and *WDR41* have not previously been reported to be associated with PD, previous findings indicate the possible associations of these genes with several neurogenerative and neurological disorders, making them strong biological candidates due to their established pleiotropy. Furthermore, variants in these genes may have utility as prognostic/diagnostic markers to stratify patients with complex (e.g., PD and other neurogenerative/neurological disorder) symptomatology. Although follow-up studies are required to confirm some findings, this study highlights the utility of genome-wide genotype imputation, followed by careful and thorough statistical analyses, in existing custom and ExomeChip array-based genetic datasets to identify intronic and intergenic risk loci, despite their sparse, inconsistent and predominantly exonic coverage.

## Figures and Tables

**Table 1:** Composition of the genotype dataset before and after imputation.

Region	MAF	Before Imputation	After Imputation	Before Imputation (%)	After Imputation (%)
Exonic	<0.05	68,026	62,829	61.56	4.29
	≥0.05	12,581	38,282	11.39	2.61
	>0	80,607	101,111	72.95	6.90
Intronic	<0.05	2385	393,633	2.16	26.85
	≥0.05	12,085	378,058	10.94	25.79
	>0	14,470	771,691	13.10	52.64
Intergenic	<0.05	3181	275,900	2.88	18.82
	≥0.05	12,246	317,236	11.08	21.64
	>0	15,427	593,136	13.96	40.46
Total	<0.05	73,592	732,362	66.60	49.96
	≥0.05	36,912	733,576	33.40	50.04
	>0	110,504	1,465,938	100	100

**Table 2:** Summary of the additional genome-wide significant loci identified after imputation.

SNP	CHR	BP	Nearest Gene	EA	NEA	EAF	MAF	OR (95% CI)	*p*-Value
**Novel PD Risk Loci**									
rs187989831	2	95,560,505	*TEKT4*	C	G	0.005	0.005	0.001 (3 × 10^−5^–0.004)	7.56 × 10^−10^
rs137887044	5	76,912,498	*WDR41*	C	T	0.972	0.028	1.85 (1.49–2.27)	2.41 × 10^−8^
rs78837976	7	100,647,511	*MUC12*	C	T	0.989	0.011	16.67 (9.09–33.33)	2.98 × 10^−18^
rs74125032	13	111,329,589	*CARS2*	T	C	0.002	0.002	4.5 × 10^−10^ (6 × 10^−13^–4 × 10^−7^)	2.36 × 10^−10^
rs117672332	17	3,606,117	*HASPIN*	T	C	0.989	0.011	7.69 (4.76–12.5)	2.20 × 10^−15^
**PD Risk Loci Reported in Other GWAS**									
rs983361	4	90,761,944	*SNCA*	T	G	0.217	0.217	0.820 (0.77–0.88)	6.29 × 10^−9^
rs7221167	17	43,933,307	*MAPT*	C	T	0.396	0.396	0.848 (0.80–0.90)	3.08 × 10^−8^

CHR = chromosome; BP = base position in GRCh37 (hg19); OR = odds ratio; EA = effect allele; NEA = non-effect allele; EAF = effect allele frequency; OR (95% CI) and *p*-value = odds ratio (95% confidence interval) and *p*-value from association analyses.

**Table 3:** Secondary association signals from conditional analysis.

Secondary SNP	CHR	BP	Nearest Gene	EA	EAF	Index SNP	*r^2^*	OR	*p*-Value	OR_cond_	*p*-Value_cond_
rs112344141	1	154,983,036	*GBA*	G	0.0491	rs35749011	0.001	1.3142	7.93 × 10^−4^	1.3337	4.18 × 10^−4^
rs113319394	2	95,555,635	*LOC442028*	C	0.0045	rs187989831	0.985	1.64 × 10^−11^	2.94 × 10^−5^	7.76 × 10^−12^	2.05 × 10^−5^
rs181580861	4	958,812	*GAK/DGKQ*	G	0.0013	rs34311866	0.0003	6.4117	4.88 × 10^−3^	7.0166	3.43 × 10^−3^
rs3806789	4	90,759,556	*SNCA*	C	0.4951	rs356182	0.174	0.9373	2.10 × 10^−2^	0.8265	9.13 × 10^−10^
rs72765119	5	76,363,276	*WDR41*	G	0.2345	rs137887044	0.0003	1.1172	2.90 × 10^−3^	1.1135	3.92 × 10^−3^
rs28645997	7	100,352,470	*MUC12*	G	0.4134	rs78837976	9.87 × 10^−5^	1.0935	2.06 × 10^−3^	1.0870	4.26 × 10^−3^
rs74125084	13	111,372,680	*CARS2*	T	0.0058	rs74125032	0.499	1.058 × 10^−4^	9.63 × 10^−8^	1.31 × 10^−4^	1.05 × 10^−6^
rs11653889	17	3,627,456	*HASPIN*	A	0.0072	rs117672332	0.747	0.0499	1.13 × 10^−14^	0.0340	1.79 × 10^−10^
rs3851784	17	45,040,117	*NSF*	A	0.4376	rs117300236	0.0115	0.8860	1.68 × 10^−5^	0.9081	6.78 × 10^−4^

Secondary SNP = secondary association single-nucleotide polymorphism; CHR = secondary SNP chromosome; BP = secondary SNP base position in GRCh37 (hg19); EA = secondary SNP effect allele; EAF = secondary SNP effect allele frequency; Index SNP = most significant SNP used to condition on; *r^2^* = LD between the secondary and index SNP; OR = odds ratio and *p*-value = *p*-value for the secondary SNP from standard association analysis; OR_cond_ = odds ratio and *p*-value_cond_ = *p*-value for the secondary SNP from conditional analyses.

**Table 4:** Comparison of association results with Nalls et al. for SNPs not available in NeuroX dataset.

SNP Information						Nalls et al. Results				Reanalysis of NeuroX Dataset		
						Discovery Phase		Replication Phase				
SNP	CHR	BP	Nearest Gene	EA	EAF	OR	*p*-Value	OR	*p*-Value	Imp_rsq	OR	*p*-Value
rs35749011	1	155,135,036	*GBA*-*SYT11*	A	0.017	1.762	6.09 × 10^−23^	2.307 *	7.48 × 10^−9^ *	0.969	2.241	5.03 × 10^−11^
rs1474055	2	169,110,394	*STK39*	T	0.128	1.213	7.12 × 10^−16^	1.218 *	1.07 × 10^−6^ *	0.961	1.241	2.82 × 10^−7^
rs115185635	3	87,520,857	*KRT8P25*	C	0.035	1.789	2.18 × 10^−8^	0.931 *	0.846 *	0.999	0.983	0.802
rs117896735	10	121,536,327	*INPP5F*	A	0.014	1.767	1.21 × 10^−11^	1.404 *	1.10 × 10^−3^ *	0.776	1.525	4.64 × 10^−4^
rs3793947	11	83,544,472	*DLG2*	A	0.443	0.912	2.59 × 10^−8^	0.976 *	0.201 *	0.998	0.983	0.538
rs11158026	14	55,348,869	*GCH1*	T	0.335	0.889	7.13 × 10^−11^	0.948	0.039	0.999	1.048	0.186
rs1555399	14	67,984,370	*TMEM229B*	A	0.468	0.872	5.53 × 10^−16^	0.971 *	0.144 *	0.902	1.033	0.239
rs62120679	19	2,363,319	*SPPL2B*	T	0.314	1.141	2.53 × 10^−9^	0.999 *	0.518 *	0.919	1.074	0.031
rs8118008	20	3,168,166	*DDRGK1*	A	0.657	1.111	2.32 × 10^−8^	1.113 *	1.18 × 10^−4^ *	0.955	1.120 *	1.13 × 10^−4^ *

CHR = chromosome; BP = base position relative in GRCh37 (hg19); EA = effect allele; EAF = effect allele frequency; OR = odds ratio and *p*-value = *p*-value of the association analysis; Imp_rsq = IMPUTE4 info score. In replication phase of Nalls et al. results, * indicates the SNPs that failed assay design or quality control and a suitable proxy SNP was used (proxy rs71628662 for rs35749011; proxy rs1955337 for rs1474055; proxy rs62267708 for rs115185635; proxy rs118117788 for rs117896735; proxy rs12283611 for rs3793947; proxy rs1077989 for rs1555399; proxy rs10402629 for rs62120679; proxy rs55785911 for rs8118008). In current study results, for rs8118008 that is not available in HRC to impute, a perfect (*r*^2^ = 1) proxy SNP rs8125675 was selected. SNPs with divergent replication results are shown in bold.

**Table 5:** GenEpi SNP–SNP interaction results.

SNP1		SNP2		Genotype freq	Nearest Gene	OR	*p*-Value
rsID	Chr:bp_Genotype	rsID	Chr:bp_Genotype				
rs11248057	4:906131_GG	rs11734449	4:921733_CC	0.101	*GAK*	1.412	4.70 × 10^−7^
rs6599388	4:939087_TT	rs1051613	4:951179_GG	0.096	*TMEM175*	1.431	3.01 × 10^−7^
rs356167	4:90673770_GG	rs34320254	4:90705606_TT	0.478	*SNCA*	0.771	1.54 × 10^−6^
rs2965400	7:21733475_GG	rs6461595	7:21758045_GG	0.132	*DNAH11*	0.750	3.77 × 10^−6^
rs2521819	17:43543830_TC	rs7224890	17:43548778_GC	0.299	*PLEKHM1*	1.260	5.55 × 10^−6^
rs34186148	17:43854655_CC	rs242941	17:43892520_CC	0.120	*CRHR1*	0.576	4.78 × 10^−10^
rs1294776	17:44004442_TT	rs6503453	17:44062603_AA	0.296	*MAPT*	0.798	9.25 × 10^−6^
rs200403	17:44781143_CA	rs35937770	17:44808360_GG	0.205	*NSF*	0.752	1.57 × 10^−7^

Chr:bp_genotype = chromosome and base position (GRCh37 [hg19]) with the genotype of each SNP; Genotype freq = frequency in all individuals (cases and controls) for the combination of SNP1 genotype and SNP2 genotype; OR = odds ratio and *p*-value = *p*-value for the interaction for each genotype combination.

## Data Availability

The individual-level PD case-control genotype data were obtained from dbGaP (https://www.ncbi.nlm.nih.gov/gap, accession number phs000918.v1.p1).

## Supplementary Materials

The following are available online at https://www.mdpi.com/article/10.3390/genes12050689/s1, Figure S1: LocusZoom plots of genome-wide significant PD loci having significant secondary association signals, Figure S2: Q-Q plot for the association results of genotype data (a) without imputation and (b) with imputation, Table S1: Composition of the initial PD-NuroX sample, Table S2: Summary of QC process, Table S3: Summary of the GWAS results for genome-wide significant loci, Table S4: Comparison of association results with Nalls et al., Table S5: Genomic region of the SNPs in the imputed dataset, Table S6: Number of cases and controls for each genotpe combination of two SNPs found in interaction analysis, Data S1: ‘NeuroX_Reanalysis_Summary_Statistics.txt’, Note S1: ‘Description_NeuroX_Reanalysis_Summary_Statistics.txt’.

## References

[1] Frazer K.A., Murray S.S., Schork N.J., Topol E.J. (2009). Human genetic variation and its contribution to complex traits. Nat. Rev. Genet..

[2] Huang J., Howie B., McCarthy S., Memari Y., Walter K., Min J.L., Danecek P., Malerba G., Trabetti E., Zheng H.-F. (2015). Improved imputation of low-frequency and rare variants using the UK10K haplotype reference panel. Nat. Commun..

[3] Chang Y.-C., Wu J.-T., Hong M.-Y., Tung Y.-A., Hsieh P.-H., Yee S.W., Giacomini K.M., Oyang Y.-J., Chen C.-Y., Weiner M.W. (2020). GenEpi: Gene-based epistasis discovery using machine learning. BMC Bioinform..

[4] de Lau L.M.L., Breteler M.M.B. (2006). Epidemiology of Parkinson’s disease. Lancet Neurol..

[5] Nalls M.A., Blauwendraat C., Vallerga C.L., Heilbron K., Bandres-Ciga S., Chang D., Tan M., Kia D.A., Noyce A.J., Xue A. (2019). Identification of novel risk loci, causal insights, and heritable risk for Parkinson’s disease: A meta-analysis of genome-wide association studies. Lancet Neurol..

[6] Nalls M.A., Pankratz N., Lill C.M., Do C.B., Hernandez D.G., Saad M., DeStefano A.L., Kara E., Bras J., Sharma M. (2014). Large-scale meta-analysis of genome-wide association data identifies six new risk loci for Parkinson’s disease. Nat. Genet..

[7] Nalls M.A., Bras J., Hernandez D.G., Keller M.F., Majounie E., Renton A.E., Saad M., Jansen I., Guerreiro R., Lubbe S. (2015). NeuroX, a fast and efficient genotyping platform for investigation of neurodegenerative diseases. Neurobiol. Aging.

[8] Purcell S., Neale B., Todd-Brown K., Thomas L., Ferreira M.A.R., Bender D., Maller J., Sklar P., de Bakker P.I.W., Daly M.J. (2007). PLINK: A tool set for whole-genome association and population-based linkage analyses. Am. J. Hum. Genet..

[9] Anderson C.A., Pettersson F.H., Clarke G.M., Cardon L.R., Morris A.P., Zondervan K.T. (2010). Data quality control in genetic case-control association studies. Nat. Protoc..

[10] Zhao S., Jing W., Samuels D.C., Sheng Q., Shyr Y., Guo Y. (2018). Strategies for processing and quality control of Illumina genotyping arrays. Brief. Bioinform..

[11] Coleman J.R., Euesden J., Patel H., Folarin A.A., Newhouse S., Breen G. (2016). Quality control, imputation and analysis of genome-wide genotyping data from the Illumina HumanCoreExome microarray. Brief. Funct. Genom..

[12] Das S., Forer L., Schönherr S., Sidore C., Locke A.E., Kwong A., Vrieze S.I., Chew E.Y., Levy S., McGue M. (2016). Next-generation genotype imputation service and methods. Nat. Genet..

[13] Danecek P., Auton A., Abecasis G., Albers C.A., Banks E., DePristo M.A., Handsaker R.E., Lunter G., Marth G.T., Sherry S.T. (2011). The variant call format and VCFtools. Bioinformatics.

[14] Rosenbloom K.R., Armstrong J., Barber G.P., Casper J., Clawson H., Diekhans M., Dreszer T.R., Fujita P.A., Guruvadoo L., Haeussler M. (2015). The UCSC Genome Browser database: 2015 update. Nucleic Acids Res..

[15] Blauwendraat C., Heilbron K., Vallerga C.L., Bandres-Ciga S., von Coelln R., Pihlstrom L., Simon-Sanchez J., Schulte C., Sharma M., Krohn L. (2019). Parkinson’s disease age at onset genome-wide association study: Defining heritability, genetic loci, and alpha-synuclein mechanisms. Mov. Disord..

[16] Chang D., Nalls M.A., Hallgrímsdóttir I.B., Hunkapiller J., van der Brug M., Cai F., Kerchner G.A., Ayalon G., Bingol B., Sheng M. (2017). A meta-analysis of genome-wide association studies identifies 17 new Parkinson’s disease risk loci. Nat. Genet..

[17] Gaare J.J., Nido G., Dölle C., Sztromwasser P., Alves G., Tysnes O.-B., Haugarvoll K., Tzoulis C. (2020). Meta-analysis of whole-exome sequencing data from two independent cohorts finds no evidence for rare variant enrichment in Parkinson disease associated loci. PLoS ONE.

[18] Auer P.L., Johnsen J.M., Johnson A.D., Logsdon B.A., Lange L.A., Nalls M.A., Zhang G., Franceschini N., Fox K., Lange E.M. (2012). Imputation of exome sequence variants into population- based samples and blood-cell-trait-associated loci in African Americans: NHLBI GO Exome Sequencing Project. Am. J. Hum. Genet..

[19] Johnsen J.M., Auer P.L., Morrison A.C., Jiao S., Wei P., Haessler J., Fox K., McGee S.R., Smith J.D., Carlson C.S. (2013). Common and rare von Willebrand factor (VWF) coding variants, VWF levels, and factor VIII levels in African Americans: The NHLBI Exome Sequencing Project. Blood.

[20] Du M., Auer P.L., Jiao S., Haessler J., Altshuler D., Boerwinkle E., Carlson C.S., Carty C.L., Chen Y.-D.I., Curtis K. (2014). Whole-exome imputation of sequence variants identified two novel alleles associated with adult body height in African Americans. Hum. Mol. Genet..

[21] Amick J., Tharkeshwar A.K., Amaya C., Ferguson S.M. (2018). WDR41 supports lysosomal response to changes in amino acid availability. Mol. Biol. Cell.

[22] Herold C., Hooli B.V., Mullin K., Liu T., Roehr J.T., Mattheisen M., Parrado A.R., Bertram L., Lange C., Tanzi R.E. (2016). Family-based association analyses of imputed genotypes reveal genome-wide significant association of Alzheimer’s disease with OSBPL6, PTPRG, and PDCL3. Mol. Psychiatry.

[23] Stein J.L., Hibar D.P., Madsen S.K., Khamis M., McMahon K.L., de Zubicaray G.I., Hansell N.K., Montgomery G.W., Martin N.G., Wright M.J. (2011). Discovery and replication of dopamine-related gene effects on caudate volume in young and elderly populations (N=1198) using genome-wide search. Mol. Psychiatry.

[24] Wolking S., Schulz H., Nies A.T., McCormack M., Schaeffeler E., Auce P., Avbersek A., Becker F., Klein K.M., Krenn M. (2020). Pharmacoresponse in genetic generalized epilepsy: A genome-wide association study. Pharmacogenomics.

[25] Nuytemans K., Maldonado L., Ali A., John-Williams K., Beecham G.W., Martin E., Scott W.K., Vance J.M. (2016). Overlap between Parkinson disease and Alzheimer disease in ABCA7 functional variants. Neurol. Genet..

[26] Linck R., Fu X., Lin J., Ouch C., Schefter A., Steffen W., Warren P., Nicastro D. (2014). Insights into the structure and function of ciliary and flagellar doublet microtubules: Tektins, Ca2+-binding proteins, and stable protofilaments. J. Biol. Chem..

[27] Hallmann K., Zsurka G., Moskau-Hartmann S., Kirschner J., Korinthenberg R., Ruppert A.-K., Ozdemir O., Weber Y., Becker F., Lerche H. (2014). A homozygous splice-site mutation in *CARS2* is associated with progressive myoclonic epilepsy. Neurology.

[28] Coughlin C.R., Scharer G.H., Friederich M.W., Yu H.-C., Geiger E.A., Creadon-Swindell G., Collins A.E., Vanlander A.V., Coster R.V., Powell C.A. (2015). Mutations in the mitochondrial cysteinyl-tRNA synthase gene, *CARS2*, lead to a severe epileptic encephalopathy and complex movement disorder. J. Med. Genet..

[29] Son A.Y., Biagioni M.C., Kaminski D., Gurevich A., Stone B., Di Rocco A. (2016). Parkinson’s Disease and Cryptogenic Epilepsy. Case Rep. Neurol. Med..

[30] Gruntz K., Bloechliger M., Becker C., Jick S.S., Fuhr P., Meier C.R., Rüegg S. (2018). Parkinson disease and the risk of epileptic seizures. Ann. Neurol..

[31] Scorza F.A., de Almeida A.-C.G., Fiorini A.C., Scorza C.A., Finsterer J. (2018). Parkinson’s disease, epileptic seizures, and sudden death: Three faces of the same coin. Epilepsy Behav..

[32] Bandres-Ciga S., Ahmed S., Sabir M.S., Blauwendraat C., Adarmes-Gómez A.D., Bernal-Bernal I., Bonilla-Toribio M., Buiza-Rueda D., Carrillo F., Carrión-Claro M. (2019). The Genetic Architecture of Parkinson Disease in Spain: Characterizing Population-Specific Risk, Differential Haplotype Structures, and Providing Etiologic Insight. Mov. Disord..

[33] Lill C.M., Roehr J.T., McQueen M.B., Kavvoura F.K., Bagade S., Schjeide B.M., Schjeide L.M., Meissner E., Zauft U., Allen N.C. (2012). Comprehensive research synopsis and systematic meta-analyses in Parkinson’s disease genetics: The PDGene database. PLoS Genet..

[34] Blauwendraat C., Reed X., Krohn L., Heilbron K., Bandres-Ciga S., Tan M., Gibbs J.R., Hernandez D.G., Kumaran R., Langston R. (2020). Genetic modifiers of risk and age at onset in GBA associated Parkinson’s disease and Lewy body dementia. Brain.

[35] Guerreiro R., Ross O.A., Kun-Rodrigues C., Hernandez D.G., Orme T., Eicher J.D., Shepherd C.E., Parkkinen L., Darwent L., Heckman M.G. (2018). Investigating the genetic architecture of dementia with Lewy bodies: A two-stage genome-wide association study. Lancet Neurol..

[36] Simón-Sánchez J., Schulte C., Bras J.M., Sharma M., Gibbs J.R., Berg D., Paisan-Ruiz C., Lichtner P., Scholz S.W., Hernandez D.G. (2009). Genome-wide association study reveals genetic risk underlying Parkinson’s disease. Nat. Genet..

[37] Nalls M.A., Plagnol V., Hernandez D.G., Sharma M., Sheerin U.M., Saad M., Simón-Sánchez J., Schulte C., Lesage S., Sveinbjörnsdóttir S. (2011). Imputation of sequence variants for identification of genetic risks for Parkinson’s disease: A meta-analysis of genome-wide association studies. Lancet.

[38] Jun G., Ibrahim-Verbaas C.A., Vronskaya M., Lambert J.C., Chung J., Naj A.C., Kunkle B.W., Wang L.S., Bis J.C., Bellenguez C. (2016). A novel Alzheimer disease locus located near the gene encoding tau protein. Mol. Psychiatry.

