# Supplementary material for: Imputation and Reanalysis of ExomeChip Data Identifies Novel, Conditional and Joint Genetic Effects on Parkinson’s Disease Risk
Source: Genes (Basel). 2021 May 4;12(5):689. doi: 10.3390/genes12050689 (PMC8147919; doi:10.3390/genes12050689)
Supplement: Supplementary file 1 [file genes-12-00689-s001.zip › Supplementary Figures S1-S2.pdf]

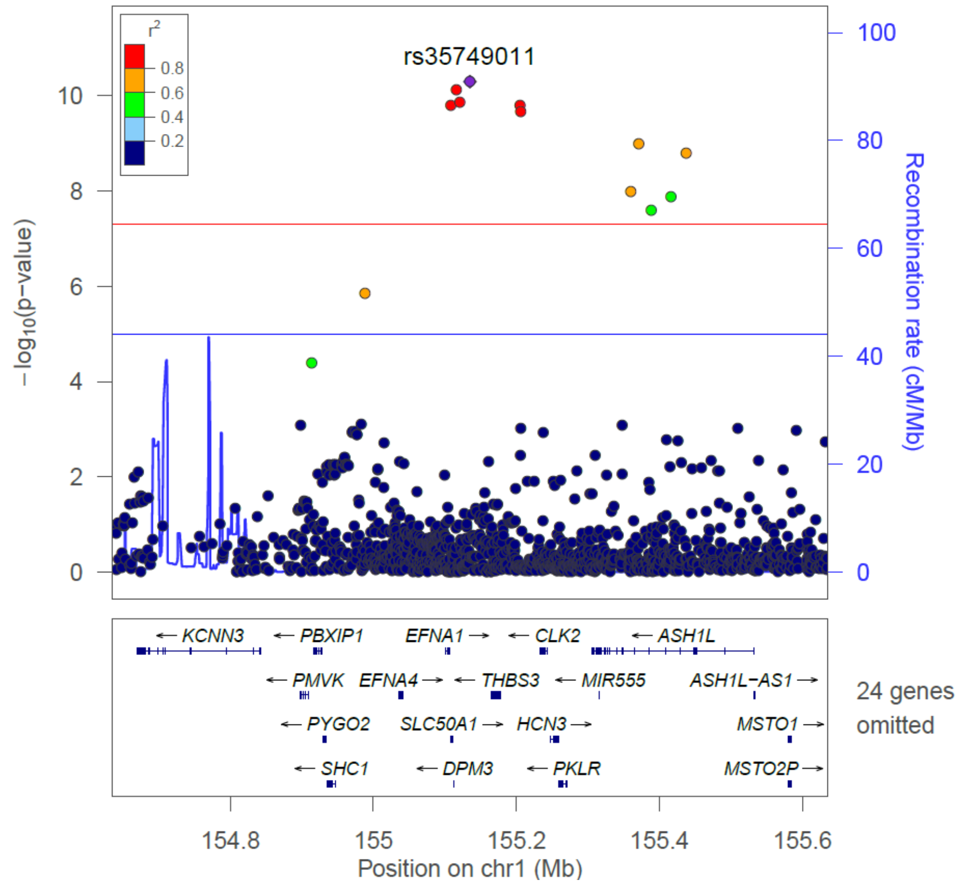
rs35749011
r2
0.8
0.6
0.4
0.2
−log10(p-value)
Recombination rate (cM/Mb)
KCNN3
PBXIP1
EFNA1
CLK2
ASH1L
PMVK
EFNA4
THBS3
MIR555
ASH1L−AS1
PYGO2
SLC50A1
HCN3
MSTO1
SHC1
DPM3
PKLR
MSTO2P
24 genes omitted
154.8
155
155.2
155.4
155.6
Position on chr1 (Mb)

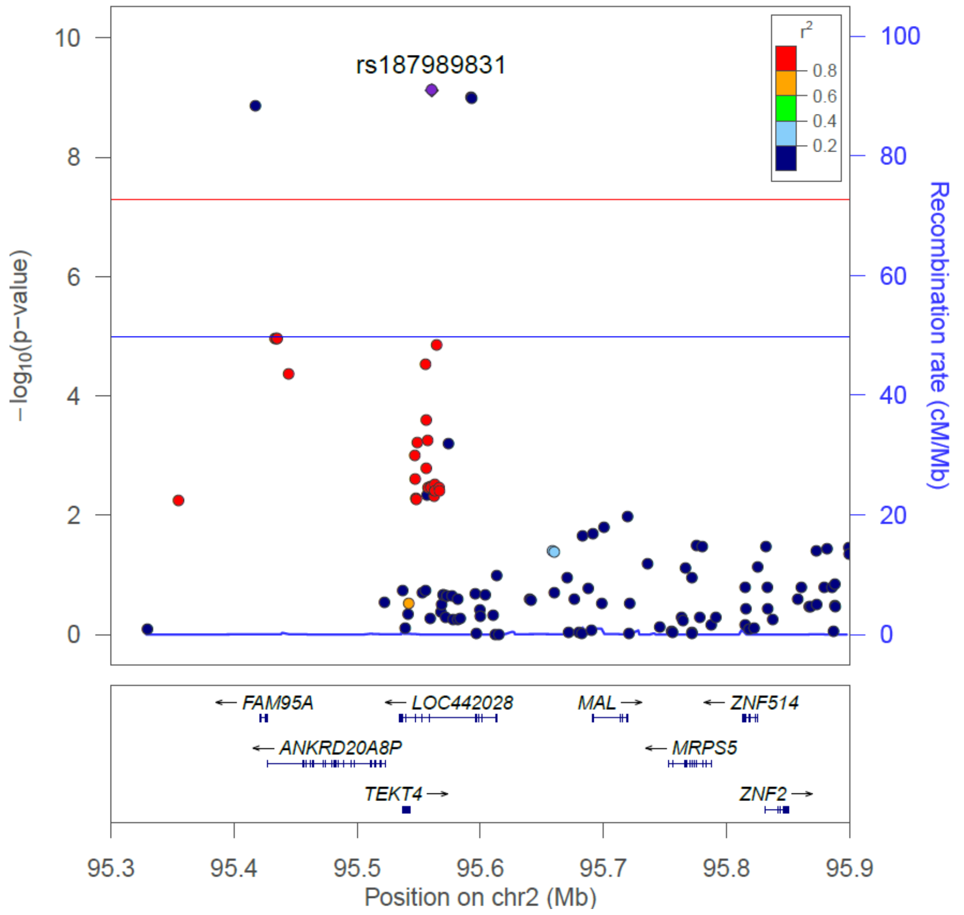
rs187989831
r2
0.8
0.6
0.4
0.2
−log10(p-value)
Recombination rate (cM/Mb)
FAM95A
LOC442028
MAL
ZNF514
ANKRD20A8P
MRPS5
TEKT4
ZNF2
95.3
95.4
95.5
95.6
95.7
95.8
95.9
Position on chr2 (Mb)

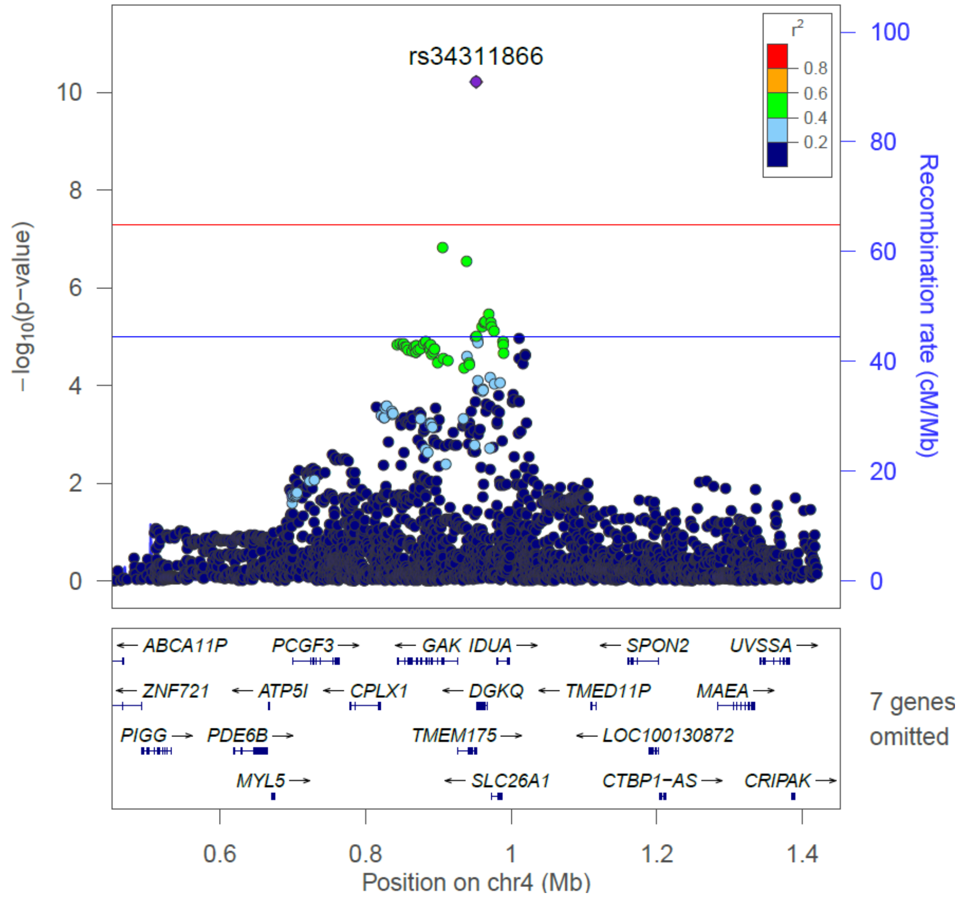
rs34311866
r²
0.8
0.6
0.4
0.2
−log10(p−value)
Recombination rate (cM/Mb)
ABCA11P
PCGF3
GAK
IDUA
SPON2
UVSSA
ZNF721
ATP5I
CPLX1
DGKQ
TMED11P
MAEA
PIGG
PDE6B
TMEM175
LOC100130872
MYL5
SLC26A1
CTBP1−AS
CRIPAK
7 genes omitted
Position on chr4 (Mb)

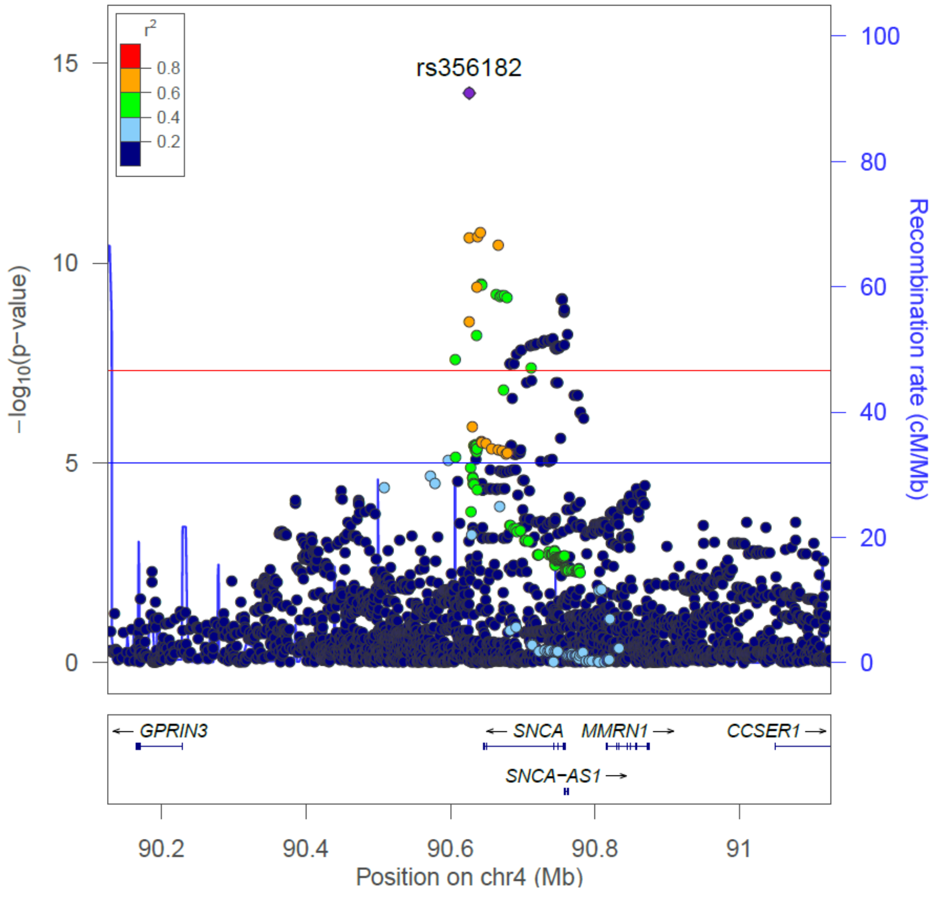
rs356182
r²
0.8
0.6
0.4
0.2
−log10(p−value)
Recombination rate (cM/Mb)
GPRIN3
SNCA
MMRN1
CCSER1
SNCA−AS1
Position on chr4 (Mb)

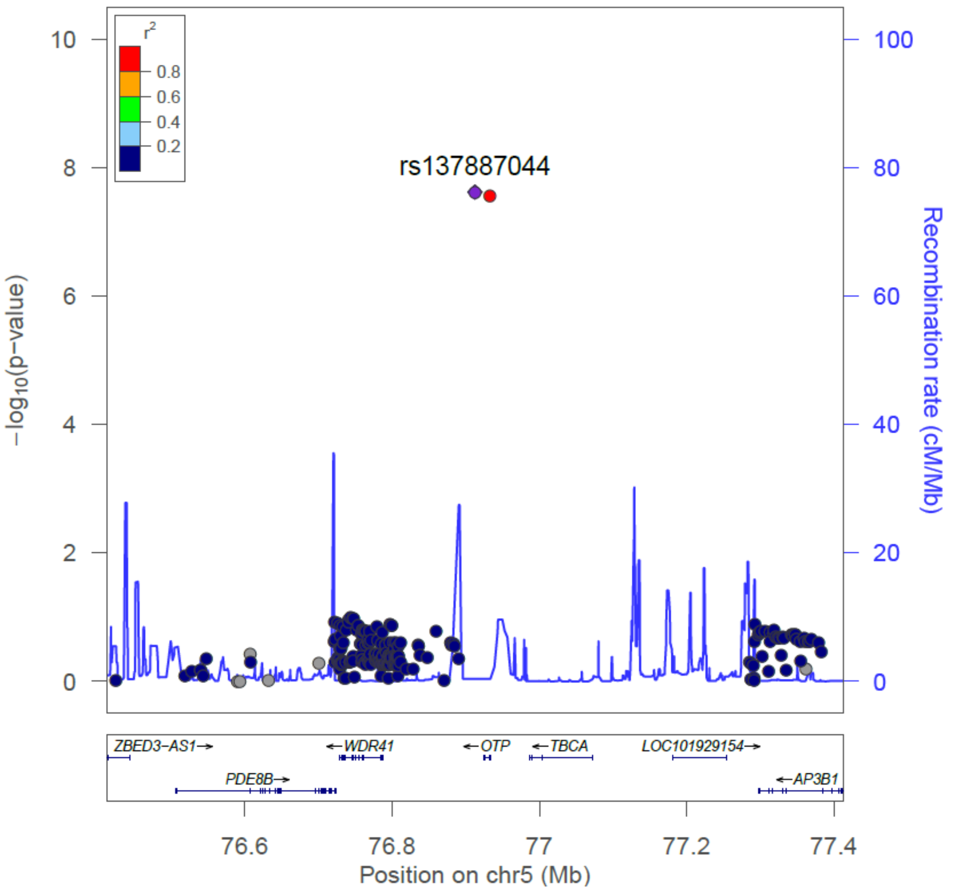

rs137887044
r2
0.8
0.6
0.4
0.2
−log10(p−value)
Recombination rate (cM/Mb)
ZBED3−AS1
WDR41
OTP
TBCA
LOC101929154
PDE8B
AP3B1
76.6
76.8
77
77.2
77.4
Position on chr5 (Mb)

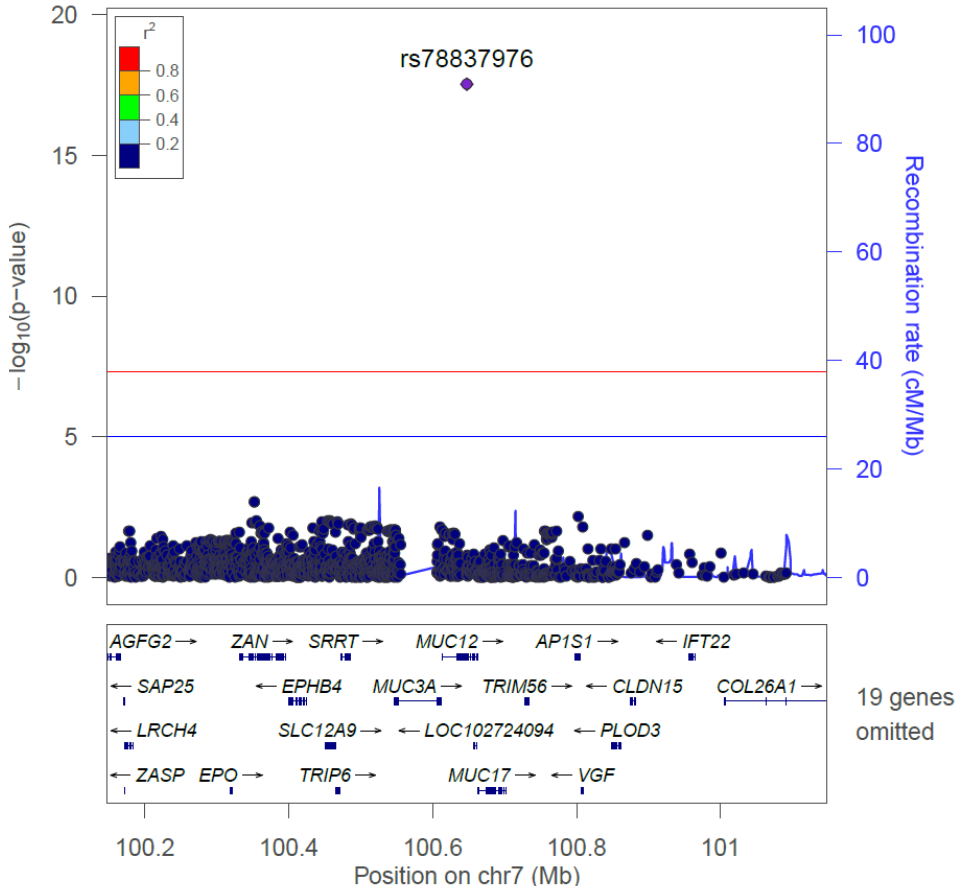

rs78837976
r2
0.8
0.6
0.4
0.2
−log10(p−value)
Recombination rate (cM/Mb)
AGFG2
ZAN
SRRT
MUC12
AP1S1
IFT22
SAP25
EPHB4
MUC3A
TRIM56
CLDN15
COL26A1
LRCH4
SLC12A9
LOC102724094
PLOD3
ZASP
EPO
TRIP6
MUC17
VGF
19 genes omitted
100.2
100.4
100.6
100.8
101
Position on chr7 (Mb)

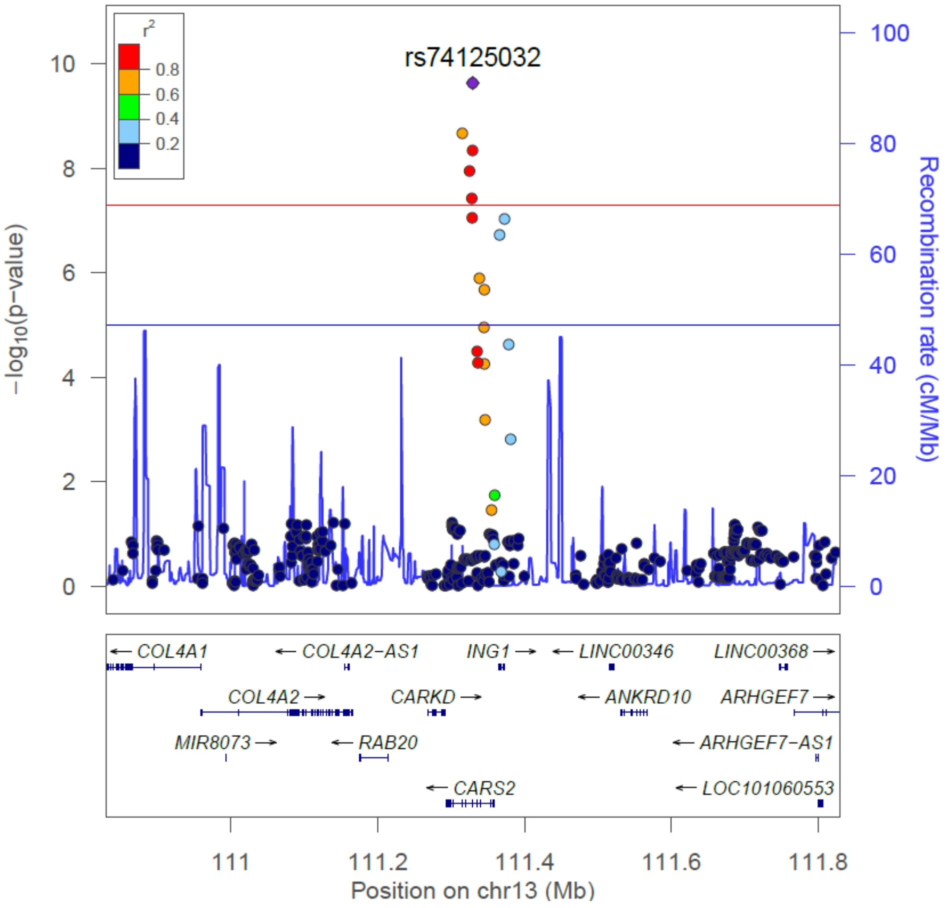
rs74125032
r2
0.8
0.6
0.4
0.2
−log10(p−value)
Recombination rate (cM/Mb)
COL4A1
COL4A2−AS1
ING1
LINC00346
LINC00368
COL4A2
CARKD
ANKRD10
ARHGEF7
MIR8073
RAB20
ARHGEF7−AS1
CARS2
LOC101060553
111
111.2
111.4
111.6
111.8
Position on chr13 (Mb)

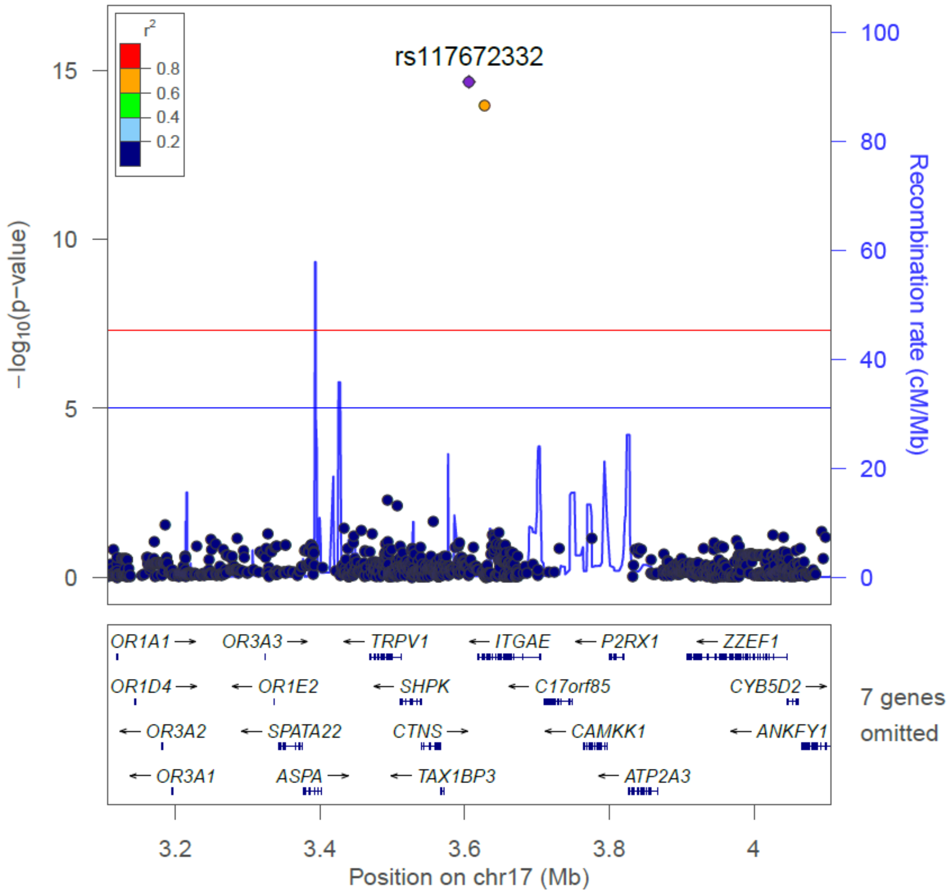
rs117672332
r2
0.8
0.6
0.4
0.2
−log10(p−value)
Recombination rate (cM/Mb)
OR1A1
OR3A3
TRPV1
ITGAE
P2RX1
ZZEF1
OR1D4
OR1E2
SHPK
C17orf85
CYB5D2
OR3A2
SPATA22
CTNS
CAMKK1
ANKFY1
OR3A1
ASPA
TAX1BP3
ATP2A3
7 genes omitted
3.2
3.4
3.6
3.8
4
Position on chr17 (Mb)

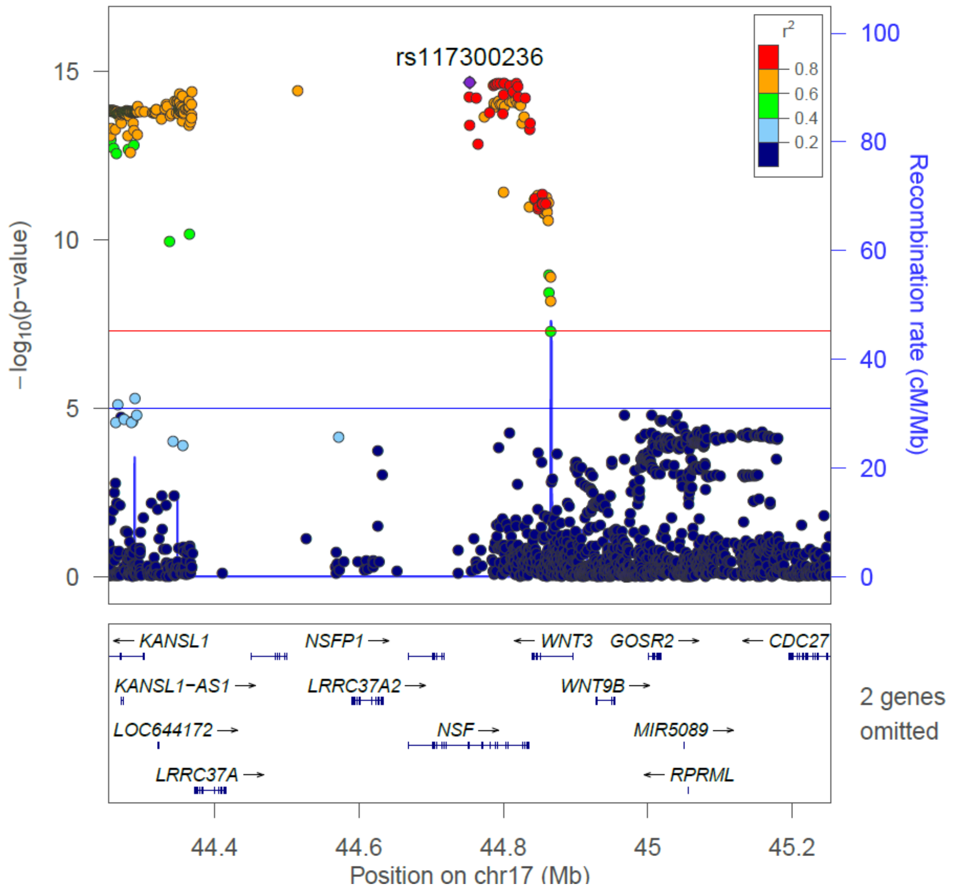

Supplementary Figure S1: LocusZoom plots of genome-wide significant PD loci having significant secondary association signals. Association significance with PD is shown as -$\log_{10}$ p-values on the left y-axis. The most significant SNP represented by purple colour diamond. All other SNPs are shown as circles and are colour coded according to the strength of LD with the most significant SNP (LD measured using the European 1000 Genomes data).

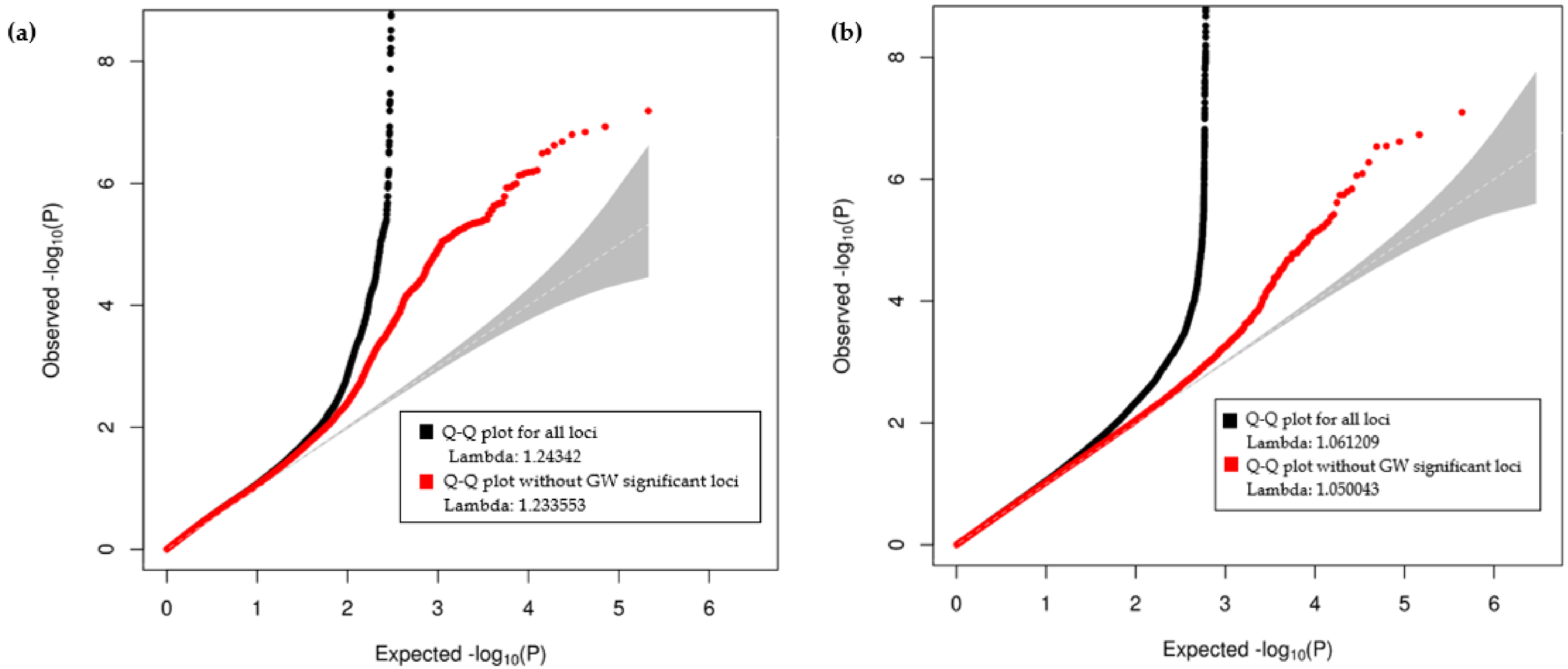

Supplementary Figure S2: Q-Q plot for the association results of genotype data (a) without imputation and (b) with imputation.
